# Morphological characteristics of subaxial cervical pedicles and surrounding critical structures in patients with vertebral artery dominance - an anatomical study based on computed tomographic imaging

**DOI:** 10.1186/s12891-022-05264-2

**Published:** 2022-03-30

**Authors:** Jin Yang, Tao Li, Qing Wang, Gaoju Wang, Song Wang, Shuang Xu, Shuai Zhang, Qiuhan Li

**Affiliations:** 1 Department of Orthopedics Surgery, Affiliated Hospital of Southwest Medical University, 25 Taiping Road, Luzhou, 646000 Sichuan China; 2 Department of Orthopedics, Zigong Fourth People’s Hospital, 19 Tan Mulin Street, Zigong, 643000 Sichuan China; 3 Department of Clinical skills center, Affiliated Hospital of Southwest Medical University, 25 Taiping Road, Luzhou, 646000 Sichuan China

**Keywords:** Imaging anatomy, Morphological characteristics, Subaxial cervical pedicle, Vertebral artery dominance, Cervical pedicle screw insertion, Vertebral artery injury

## Abstract

**Background:**

No study has assessed the feasibility and safety of cervical pedicle screw implantation in patients with vertebral artery dominance (VAD), a common vertebral artery (VA) variation which can increase VA injury (VAI) risk. This study was to assess morphological characteristics of the subaxial cervical pedicles and surrounding critical structures, and identify their correlations in patients with VAD.

**Methods:**

Computed tomography arteriography scans of 152 patients were used for retrospectively measuring parameters including pedicle outer width (POW), the distance from the lateral pedicle border to the closest part of VA (DPVA), diameter of VA (DVA), area of VA (AVA), area of transverse foramen (ATF) and occupational ratio of transverse foramen (TF). Moreover, correlations among some critical parameters were assessed.

**Results:**

One hundred eight males and 44 females, with a mean age of 55.9 years were included. POW was smaller on the dominant side than on the non-dominant side, whereas DPVA, DVA, AVA, ATF, and TF were larger on the dominant side than those on the non-dominant side. On both sides, POW < 4 mm and POW + DPVA < 5 mm were observed most frequently at C3 and C4. On both sides, POW was correlated to ATF, and ATF was correlated to DVA and AVA. DPVA was correlated to ATF on the dominant side.

**Conclusion:**

Patients with VAD exhibited smaller POW on the dominant side, most frequently at C3 and C4. Dominant VA may indirectly affect POW. TF may be a key determinant of DPVA and POW.

## Background

Cervical pedicle screw (CPS) fixation, first introduced by Abumi [1], provides stronger biomechanical stability than other cervical internal fixation techniques for treating spinal deformity, severe fracture and dislocation, and for multiple-level reconstruction [2–4]. However, CPS fixation can be technically challenging due to the anatomical complexity of the pedicle region and potential risk of vertebral artery (VA) and spinal cord injury, limiting its application.

Vertebral artery injury (VAI) is one of the major concerns during CPS fixation. Although VAI is uncommon in cervical spine surgeries [5–9], the consequences can be catastrophic, including fistulas, pseudoaneurysm, late-onset hemorrhage, thrombosis, embolism, cerebral ischemia, and death [10, 11]. The risks of VAI are closely related to the anatomical characteristics of the cervical pedicle and surrounding structures and to the condition of the pedicle insertion [12]. Therefore, a better understanding of these morphological characteristics is essential to reduce the risk of VAI during CPS placement surgery. Furthermore, anatomical variation of the cervical spine may increase the risk of neurovascular injury. Vertebral artery dominance (VAD) is a common variation with a reported prevalence from 38.5 to 73% according to different definitions [13–15]. The dominant vertebral artery is the main blood supply to the brainstem and epencephalon, so the consequences of injury are even more severe than injury to the normal VA [14, 16].

Given the frequency of VAD, it is critical to assess methods for safer and more efficacious CPS placement in patients with VAD. To our knowledge, however, no study has focused on the morphological characteristics of the subaxial cervical pedicle and surrounding structures just in patients with VAD. Therefore, we conducted a morphological analysis of these characteristics based on computed tomographic arteriography (CTA) and analyzed correlations among these various structures.

## Materials and methods

### Study population

**Fig. 1:**
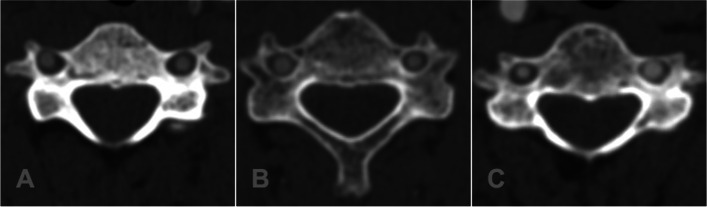
**A** Normal vertebral artery; **B** Diameter difference between bilateral vertebral arteries ≤0.8 mm which did not meet our inclusion criteria; **C** Diameter difference between bilateral vertebral arteries ≥0.8 mm included in this study

Our hospital ethics committee approved this retrospective image-based study. We reviewed 697 patients who underwent CTA at our hospital from January 2012 to March 2019. Based on the findings of previous studies [13, 17], we defined VAD as a diameter difference between bilateral vertebral arteries ≥0.8 mm (Fig. 1). As the aim was to explore morphological characteristics of the subaxial cervical pedicle and surrounding structures in patients with VAD, the subaxial cervical spine in this study was defined as from C3 to C6.

Patient inclusion criteria were 1) VAD, 2) computed tomography (CT) scan range C1–C7, 3) CT scanning using 256-slice CT, 4) age ≥ 18 and ≤ 60 years, and 5) complete clinical and imaging data. Patient exclusion criteria were 1) severe cervical fracture and dislocation (dislocation > 1/2 the anterior-posterior diameter of the lower vertebral body), 2) severe cervical deformity such as kyphosis > 20 degrees, scoliosis > 10 degrees, basilar invagination, or Chiari malformation, 3) ankylosing spondylitis, 4) diffuse idiopathic skeletal hyperostosis, 5) cervical spine surgery history, 6) VA embolization, VA atherosclerosis, or a single VA, and 7) other diseases such as tumor or severe osteoporosis.

### Measurements

All measurements were performed on a CT advanced workstation 4.4 (GE, USA). Scan parameter settings were as follows: layer thickness 0.625 mm, layer spacing 0.625 mm, window width 1300 HU, and window level 400 HU. Measurement accuracy was ±0.1 mm. Measurements were performed on the axial CT image showing the maximum pedicle outer width; due to the maximum pedicle outer width shown by this image, it was considered that this image presented the center of the pedicle or reflected the content at the center of the pedicle. Furthermore, the adjacent upper axial CT image of that one and lower one were also measured for all parameters and the data measured from the three images were averaged for analysis. Multiple-planar reconstruction was used to conduct multiple-point observation and identification for accurate measurement.

**Fig. 2:**
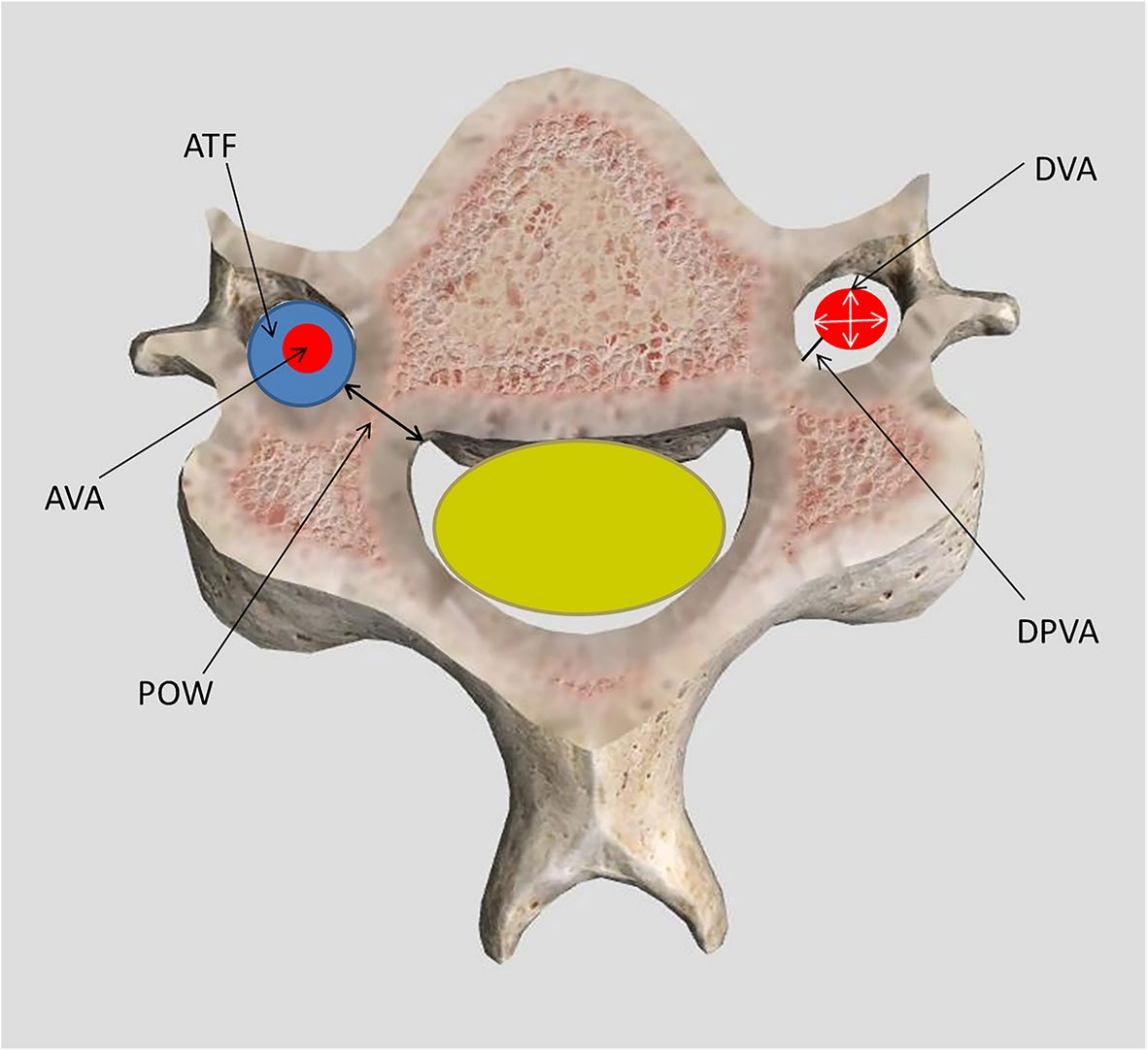
A schematic representation of the measuring parameters. POW, the distance between the lateral cortical border and medial border at the most stenotic area of the pedicle; DPVA, the distance from the lateral pedicle border to the closest part of the vertebral artery (VA); DVA, diameter of the VA; AVA, area of VA; ATF, area of transverse foramen

The following parameters were measured or calculated from C3–C6 bilaterally: 1) POW, the distance between the lateral cortical border and medial border at the most stenotic area of the pedicle; 2) DPVA, the distance from the lateral pedicle border to the closest part of VA; 3) DVA, diameter of the VA, for oval artery, the diameter was defined as the average of the longest axis distance and the shortest one of the VA; 4) AVA, area of VA, was calculated according to DVA (AVA = π × (DVA /2)^2^; 5) ATF, area of transverse foramen (TF), was calculated by the method similar to AVA; 6) ORTF, occupation ratio of TF (ORTF = AVA/ATF) (Fig. 2). All parameters were measured and/or calculated thrice at separate times by two independent readers, a spinal surgeon and senior imaging diagnostician.

### Statistical analysis

Statistical analysis was conducted using SPSS version 24.0 (IBM Corp., Armonk, NY, USA). The reliability of measurement data was tested using the intraclass correlation coefficient (ICC); ICC ≥ 0.75 was considered to show good reliability. If the data were found to have good reliability, the average value of data was subsequently adopted for statistical analysis.

The Shapiro-Wilk test was used to test the normality of all datasets. Continuous data are presented as mean and standard deviation (SD) when normally distributed or as median and interquartile range (IQR) when not normally distributed. Continuous parametric data were compared using Student’s t test and categorical data by χ^2^ test or Fisher’s exact test. Pearson’s correlation coefficient was used to evaluate the quantitative associations among parameters. A *P* <  0.05 (two-tailed) was considered significant for all tests.

## Results

### Demographic characteristics

Based on our inclusion and exclusion criteria, 152 patients with VAD (21.8%, 152/697) were enrolled, including 108 males (71.1%) and 44 females (28.9%) of mean age 55.9 (SD, 7.6) years, mean body weight of 57.2 (SD, 8.0) kg, and median height of 167 (IQR, 160–172) cm. Most cases with VAD (69.1%) were on the left side. Twenty cases (13.2%) exhibited VA pathway variation, with VA entry into the TF at C5, and 85% of these cases had a nearly closed TF. Therefore, DPVA and ATF were not measured at C6. Ten cases (6.6%) did not have an intact TF, including two cases with incomplete TF at C3, one at C4, five at C5, and ten at C6. Therefore, ATF was also not measured at these levels. All data of these 30 cases were excluded for correlational analyzes. Detailed demographic information is summarized in Table 1.

**Table 1:** Demographic characteristics of 152 Patients

	Mean and standard deviation or Median (Interquartile Range) or n (%)
Age (years)	55.9 ± 7.6
Sex, male(%)	108(71.1)
Height	167(160–172)
Body weight	57.2 ± 8.0
Side of dominant artery	
Left	105(69.1)
Right	47(30.9)
Diagnosis or Diseases	
Cervical spondylotic radiculopathy	11(7.2)
Cervical spondylotic myelopathy	107(70.3)
Cervical SCI without fracture and dislocation	10(6.6)
Cervical fracture	11(7.2)
Cervical tuberculosis	8 (5.3)
Posterior circulation ischemia	5(3.3)
Other deformities	
Variation in vertebral artery pathway	20 (13.2)
Incomplete transverse foramen	10(6.6)
Comorbidities	
Hypertension	5(3.3)
Coronary heart disease	2(1.3)
Diabetes	3(2.0)
Pakinson disease	3(2.0)
Chronic pulmonary disease	4(2.6)
Other fratcure	5(3.3)
Smoking	25(16.4)
Other surgery history	11(7.2)

### Measurement results

**Fig. 3:**
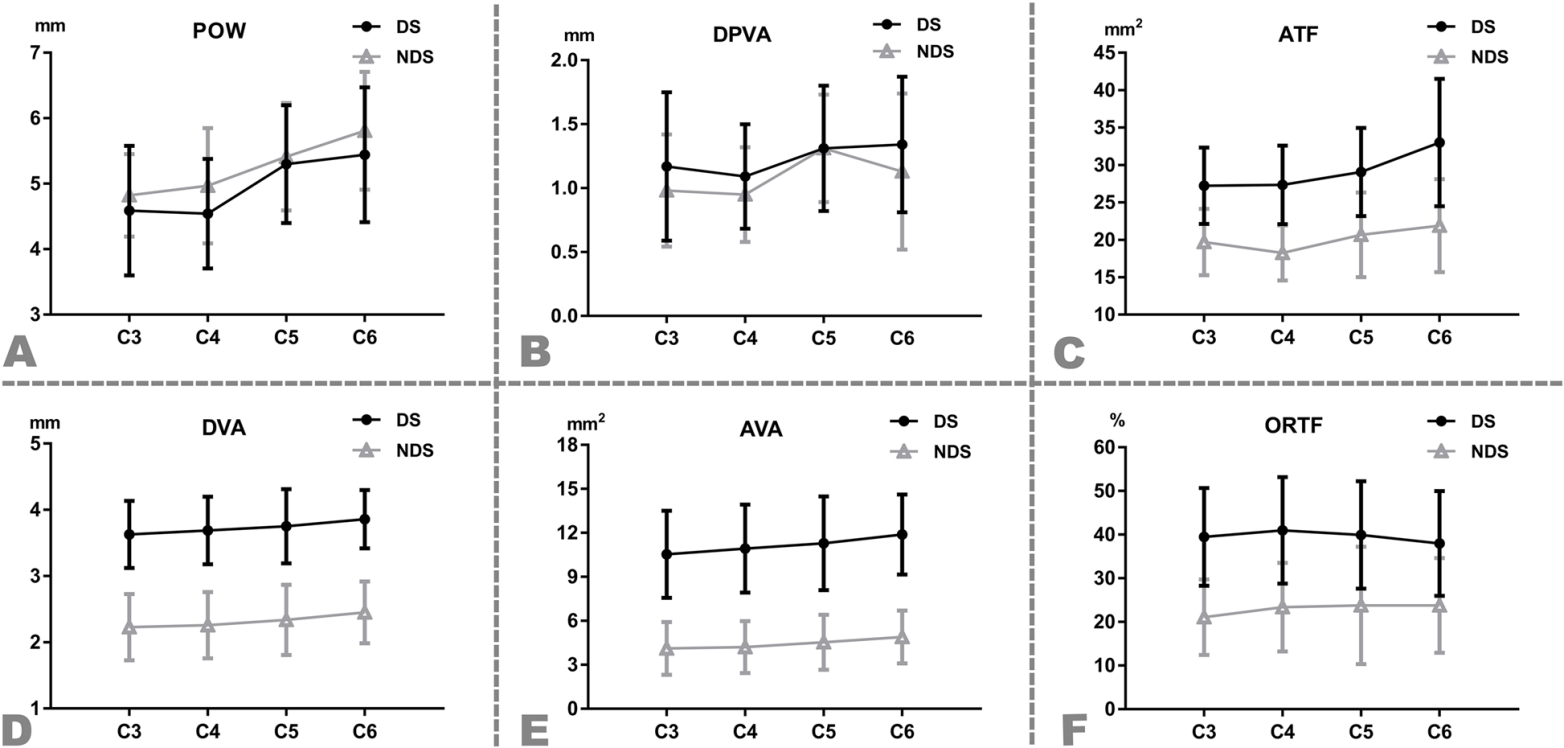
The parameters such as POW (**A**), DPVA (**B**), ATF (**C**), DVA (**D**), and AVA (**E**) exhibited a gradual increase on both sides from C3 to C6, whereas ORTF exhibited a slight decrease on the dominant side from C4 to C6. POW, pedicle outer width; DPVA, the distance from the lateral pedicle border to the closest part of the vertebral artery; ATF, area of transverse foramen; DVA, diameter of vertebral artery; AVA, and area of vertebral artery ORTF, occupation ratio of transverse foramen; DS, dominant side; NDS, non-dominant side

**Fig. 4:**
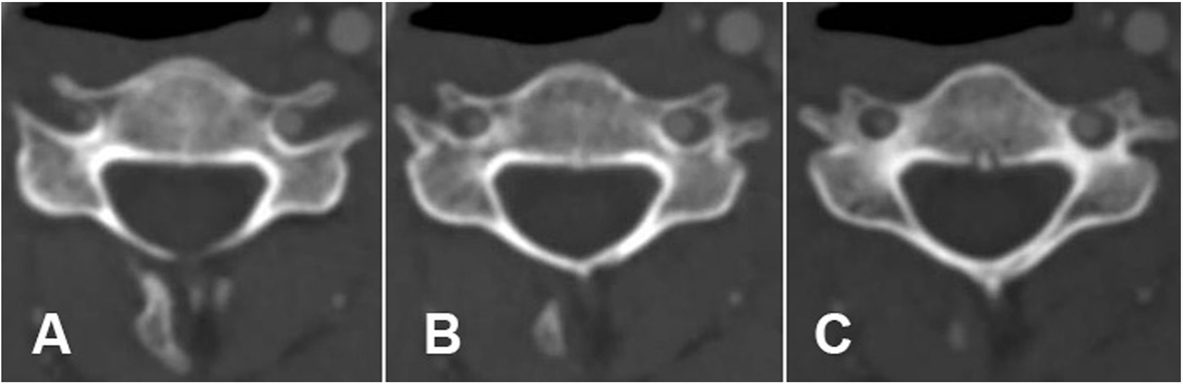
Series CT images of C4 show that the patient with VAD exhibit smaller POW on the dominant side than that on the non-dominant side (**A**: 4.43 mm VS 5.41 mm; **B**: 4.92 mm VS 5.73 mm; **C**: 4.42 mm VS 5.34 mm; Averaged POW: 4.59 mm VS 5.49 mm). VAD, vertebral artery dominance; POW, the distance between the lateral cortical border and medial border at the most stenotic area of the pedicle

The average intra-reader and inter-reader reliabilities according to the ICC were > 0.75 (good) for all parameters. The mean POW of all cases was significantly smaller on the dominant side than the non-dominant side (4.97 ± 1.02 mm vs. 5.29 ± 0.92 mm, *P* <  0.001) (A characteristic image in Fig. 4), while DPVA was significantly larger on the dominant side than the non-dominant side (1.22 ± 0.52 mm vs. 1.09 ± 0.49 mm, *P* <  0.001). However, there were no significant differences between two sides in POW and DPVA at C5 (*P* > 0.05). The DVA was also larger on the dominant side than the non-dominant side (3.73 ± 0.51 mm vs. 2.32 ± 0.51 mm, *P* <  0.001), and the bilateral difference was significant at every level (*P* <  0.001). The AVA calculated based on DVA exhibited a similar trend. The ATF was significantly larger on the dominant side than the non-dominant side (29.10 ± 6.72 mm^2^ vs. 20.09 ± 5.23 mm^2^, P <  0.001). Similarly, ORTF was significantly greater on the dominant side than the non-dominant side (39.60% ± 11.95% vs. 22.96% ± 10.65%, P <  0.001). More detailed information is presented in Table 2. All parameters except ORTF exhibited a gradual increase on both sides from C3 to C6 (Fig. 3A-E), while ORTF showed a slight decrease from C4 to C6 on the dominant side (Fig. 3F).

**Table 2:** The Comparison of all parameters between the dominant side and non-dominant side

	Dominant side		Non-dominant side		Intergroup difference		t value	*P* value
	Range	Mean ± SD	Range	Mean ± SD	Mean	95%CI		
POW (mm)								
C_3_(*n* = 152)	2.20 ~ 7.30	4.59 ± 0.99	3.90 ~ 7.10	4.82 ± 0.63	−0.23	−0.04 ~ −0.41	−2.369	0.019
C4 (*n* = 152)	2.40 ~ 6.70	4.54 ± 0.84	3.00 ~ 6.90	4.97 ± 0.88	− 0.43	− 0.24 ~ − 0.62	−4.373	< 0.001
C5 (*n* = 152)	3.20 ~ 8.20	5.30 ± 0.90	3.60 ~ 8.60	5.41 ± 0.82	− 0.11	0.08 ~ − 0.30	−1.112	0.267
C6 (*n* = 152)	2.60 ~ 7.50	5.44 ± 1.03	4.10 ~ 8.40	5.81 ± 0.90	− 0.51	−0.30 ~ − 0.73	−4.632	< 0.001
Total (*n* = 608)	2.20 ~ 8.20	4.97 ± 1.02	3.00 ~ 8.60	5.29 ± 0.92	− 0.32	− 0.21 ~ − 0.43	−5.719	< 0.001
DPVA (mm)								
C_3_(*n* = 152)	0.30 ~ 2.90	1.17 ± 0.58	0.3 ~ 2.80	0.98 ± 0.44	0.19	0.30 ~ 0.07	3.189	0.002
C_4_(*n* = 152)	0.30 ~ 2.40	1.09 ± 0.41	0.30 ~ 1.80	0.95 ± 0.37	0.13	0.22 ~ 0.04	2.948	0.003
C_5_(*n* = 152)	0.30 ~ 2.60	1.31 ± 0.49	0.30 ~ 2.90	1.31 ± 0.42	−0.01	0.10 ~ − 0.11	−0.125	0.901
C_6_(*n* = 132)	0.30 ~ 3.20	1.34 ± 0.53	0.30 ~ 4.20	1.13 ± 0.61	0.21	0.35 ~ 0.07	2.969	0.003
Total (*n* = 588)	0.30 ~ 3.20	1.22 ± 0.52	0.30 ~ 4.20	1.09 ± 0.49	0.13	0.19 ~ 0.07	4.387	< 0.001
DVA (mm)								
C_3_(*n* = 152)	2.65 ~ 5.10	3.63 ± 0.51	1.15 ~ 3.65	2.23 ± 0.50	1.40	1.51 ~ 1.28	23.94	< 0.001
C4 (*n* = 152)	2.45 ~ 4.85	3.69 ± 0.51	1.05 ~ 3.40	2.26 ± 0.50	1.44	1.55 ~ 1.32	24.61	< 0.001
C5 (*n* = 152)	2.65 ~ 4.90	3.75 ± 0.56	1.00 ~ 3.55	2.34 ± 0.53	1.41	1.53 ~ 1.29	22.65	< 0.001
C6 (*n* = 152)	3.05 ~ 5.10	3.86 ± 0.44	1.25 ~ 3.55	2.45 ± 0.47	1.41	1.51 ~ 1.31	26.93	< 0.001
Total (*n* = 608)	2.45 ~ 5.10	3.73 ± 0.51	1.00 ~ 3.65	2.32 ± 0.51	1.41	1.47 ~ 1.36	48.2	< 0.001
AVA (mm^2^)								
C_3_(*n* = 152)	5.52 ~ 20.43	10.53 ± 2.97	1.04 ~ 10.46	4.11 ± 1.80	6.43	6.98 ~ 5.87	22.83	< 0.001
C4 (*n* = 152)	4.71 ~ 18.47	10.92 ± 3.00	0.87 ~ 9.08	4.20 ± 1.78	6.72	7.28 ~ 6.17	23.77	< 0.001
C5 (*n* = 152)	5.52 ~ 18.86	11.29 ± 3.20	0.79 ~ 9.90	4.53 ± 1.88	6.76	7.35 ~ 6.17	22.45	< 0.001
C6 (*n* = 152)	7.31 ~ 20.43	11.88 ± 2.73	1.23 ~ 9.90	4.90 ± 1.80	6.98	7.51 ~ 6.46	26.33	< 0.001
Total (*n* = 608)	4.71 ~ 20.43	11.16 ± 3.01	0.79 ~ 10.46	4.43 ± 1.84	6.72	7.01 ~ 6.44	46.97	< 0.001
ATF (mm^2^)								
C_3_(*n* = 150)	18.10 ~ 46.30	27.21 ± 5.13	11.90 ~ 32.40	19.71 ± 4.44	7.51	8.60 ~ 6.41	13.54	< 0.001
C_4_(*n* = 151)	16.80 ~ 41.60	27.33 ± 5.25	10.20 ~ 28.20	18.23 ± 3.69	9.11	10.13 ~ 8.08	17.437	< 0.001
C_5_(*n* = 147)	16.90 ~ 47.50	29.07 ± 5.90	5.00 ~ 32.20	20.66 ± 5.65	8.41	9.73 ~ 7.08	12.485	< 0.001
C_6_(*n* = 142)	6.30 ~ 66.30	33.01 ± 8.54	10.80 ~ 42.20	21.89 ± 6.22	11.12	12.87 ~ 9.38	12.55	< 0.001
Total (*n* = 590)	6.30 ~ 66.30	29.10 ± 6.72	5.00 ~ 42.20	20.09 ± 5.23	9.01	9.70 ~ 8.32	25.692	< 0.001
ORTF(%)								
C_3_(*n* = 150)	15.40 ~ 80.70	39.47 ± 11.21	4.80 ~ 49.70	21.08 ± 8.66	18.39	20.66 ~ 16.11	15.897	< 0.001
C_4_(*n* = 151)	15.40 ~ 83.60	40.95 ± 12.24	3.20 ~ 60.40	23.38 ± 10.16	17.57	20.12 ~ 15.02	13.572	< 0.001
C_5_(*n* = 147)	13.90 ~ 72.90	39.91 ± 12.30	3.10 ~ 76.20	23.75 ± 13.49	15.36	18.76 ~ 11.95	11.231	< 0.001
C_6_(*n* = 142)	15.40 ~ 85.60	37.98 ± 11.98	3.60 ~ 61.20	23.77 ± 10.88	14.91	18.13 ~ 11.69	10.464	< 0.001
Total (*n* = 590)	13.90 ~ 85.60	39.60 ± 11.95	3.10 ~ 76.20	22.96 ± 10.65	16.59	18.03 ~ 15.15	25.236	< 0.001

*POW* the distance between lateral cortical border and medial border at the most stenotic area of the pedicle, *DPVA* the distance between lateral pedicle border and medial border of vertebral artery, *DVA* diameter of vertebral artery, *AVA* area of vertebral artery, *ATF* area of transverse foramen, *ORTF* occupation ratio of transverse foramen

As POW and DPVA are the key factors related to VAI risk, more detailed analyses of these parameters were conducted. A significantly greater proportion of VAD cases demonstrated POW < 4 mm on the dominant side than the non-dominant side (76 [12.5%] vs. 36 [5.9%], *P* <  0.001) (Table 3). Further, a significantly greater proportion of VAD cases exhibited POW + DPVA < 5 mm on the dominant side than the non-dominant side (79 [13.4%] vs. 49 [8.3%], *P* = 0.006) (Table 4).

**Table 3:** The number and proportion of pedicle outer width < 4 mm

	Dominant side, n (%)	Non-dominant side, n (%)	*P* value
C3 (*N* = 152)	33(21.7)	14(9.2)	0.004
C4 (*N* = 152)	28(18.4)	19(12.5)	0.204
C5 (*N* = 152)	7(4.6)	3(0.7)	0.336
C6 (*N* = 152)	8(5.3)	0	0.007
Total (*N* = 608)	76(12.5)	36(5.9)	< 0.001

**Table 4:** The number and proportion of (POW + DPVA) < 5 mm

	Dominant side, n (%)	Non-dominant side, n (%)	*P* value
C3 (*N* = 152)	32 (21.1)	20 (13.2)	0.093
C4 (*N* = 152)	31 (20.3)	22 (14.5)	0.226
C5 (*N* = 152)	7 (4.6)	2 (1.3)	0.173
C6 (*N* = 132)	9 (6.8)	5 (3.8)	0.411
Total (*N* = 588)	79(13.4)	49(8.3)	0.006

*POW* the distance between lateral cortical border and medial border at the most stenotic area of the pedicle, *DPVA* the distance from the lateral pedicle border to the closest part of vertebral artery

### Correlations

POW was not correlated with DVA (*P* = 0.060) or AVA (*P* = 0.054) on the dominant side; however, POW was correlated with DVA (r = 0.123, P = 0.006) and AVA (r = 0.132, *P* = 0.004) on the non-dominant side and with ATF on both sides. POW was also correlated with DPVA on both sides.

DPVA was not correlated with DVA or AVA on either side; however, DPVA was weakly correlated with ATF (r = 0.150, *P* = 0.001) on the dominant side but not the non-dominant side (r = 0.088, *P* = 0.053). ATF was correlated with DVA and AVA on both sides (Table 5). Correlation analysis between DVA and AVA was not conducted due to their relationship and ORTF was not included in the correlation analysis due to data characteristics.

**Table 5:** The Correlations among the parameters in the dominant and non-dominant sides

		Dominant Side		Non-Dominant Side	
		*R* value	*P* value	*R* value	*P* value
POW	DPVA	0.176	< 0.001	0.118	0.009
POW	DVA	0.085	0.060	0.123	0.006
POW	AVA	0.087	0.054	0.132	0.004
POW	ATF	0.131	0.004	0.166	< 0.001
DPVA	DVA	−0.002	0.967	−0.022	0.632
DPVA	AVA	0.000	0.992	−0.015	0.737
DPVA	ATF	0.150	0.001	0.088	0.053
DVA	ATF	0.280	< 0.001	0.260	< 0.001
AVA	ATF	0.285	< 0.001	0.264	< 0.001

*POW* the distance between lateral cortical border and medial border at the most stenotic area of the pedicle, *DPVA* the distance from the lateral pedicle border to the closest part of vertebral artery, *DVA* diameter of vertebral artery, *AVA* area of vertebral artery, *ATF* area of transverse foramen

## Discussion

We report the first morphological characterization of the pedicles and surrounding structures from C3 to C6 in patients with VAD, and analyze correlations among these structures to provide an anatomical basis for safer and more accurate CPS insertion or appropriate choice of internal fixation in this patient group. The prevalence of VAD (21.8%) was lower than in previous studies (from 38.5 to 73%) [13–15], this was possibly due to differences in inclusion criteria, exclusion criteria, or VAD definition. Most VAD (69.1%) was found on the left side, which is consistent with previous findings [18].

POW is the most important factor influencing the safety and feasibility of CPS insertion. According to several previous studies, the lateral wall is the thinnest of the cervical pedicle walls [12, 19]. Therefore, it is the easiest to breach during CPS insertion and so cause the potential risk of VAI [6–8]. In this study, POW was significantly smaller on the dominant side than the non-dominant side (*P* <  0.001). Furthermore, POW was more frequently < 4 mm on the dominant side compared to the non-dominant side (P <  0.001), indicating that difficult or unfeasible CPS insertion is more common on the dominant side in VAD patients [12, 20]. Moreover, this may confer a greater potential risk of pedicle wall breach on the dominant side than the non-dominant side in VAD patients. However, several studies reported that lateral wall breach or even mild intrusion into the TF did not result in a higher VAI rate [7, 8, 21]. We suggest that a “safe space” between the VA and pedicle lateral wall, the DPVA, may account for above mentioned findings. Additionally, DPVA is a more specific and direct value than ORTF, so we combined POW with DPVA, and examined POW + DPVA < 5 mm as an assessment parameter in further analysis. Despite larger DPVA on the dominant side (*P* <  0.001), the frequency of POW + DPVA < 5 mm was also higher on the dominant side (*P* = 0.006), suggesting that the bilateral difference in DPVA is insufficient as a metric to guide the choice of CPS insertion. Nonetheless, both POW and POW + DPVA measures suggest a higher risk of pedicle border breach and ensuing VAI on the dominant side than the non-dominant side.

POW < 4 mm was found more frequently at C3 and C4 on both sides but was most frequent on the dominant side (*P* <  0.001). Similarly, POW + DPVA < 5 mm was most common at C3 and C4, so surgeons should be more careful in choosing CPS treatment at these levels for patients with VAD. With the development and application of new surgical navigation methods, the ability to accurately identify the best CPS insertion position and angle has significantly improved [21–24]. However, smaller POW and DPVA are both indicative of VAI risk. Therefore, we do not recommend CPS insertion at pedicles with POW < 4 mm or POW + DPVA < 5 mm, especially on the dominant side due to the potential of VAI.

Despite smaller POW on the dominant side, POW showed no direct correlation with DVA or AVA on the dominant side, although it was correlated with DVA and AVA on the opposite side. POW was, however, correlated with ATF and the latter was correlated with DVA or AVA on both sides. These results suggest that POW on the dominant side is influenced directly or to a greater extent by ATF than by DVA or AVA. We speculate that VAD may indirectly affect the development of the pedicle. During the embryonic stage, the VA develops before the TF. Therefore, VAD may have a stronger influence on TF than a normal VA, and the larger TF on the dominant side may further affect the development of the ipsilateral pedicle. This indirect influence on the pedicle may not manifest as a correlation between POW and DVA or AVA. Additionally, in our study, 20 cases exhibited pathway variation for TF entry at C5, and 85% of these cases had a nearly closed TF, which supports this notion.

DPVA was correlated with ATF on the dominant side but not on the non-dominant side. This further supports the greater developmental influence of VAD. In our study, DVA, AVA, and ATF were significantly larger on the dominant side and gradually increased from C3 to C6. Additionally, the increase in ATF at lower cervical levels appeared larger on the dominant side. This tendency was reflected by a decline in ORTF (AVA/ATF) at lower levels (Fig. 3F). Further, this difference in ATF increase rate compared to both DVA and AVA would influence DPVA and POW on the dominant side. Therefore, the development of the TF may be a key factor influencing the final DPVA and POW, especially on the dominant side. In general, the correlations between all parameters were not strong; more clear correlations between them needs further study with larger sample size in the future.

## Limitations

The major limitation of this study is the retrospective design, which may introduce selection bias. Second, the study did not include severe cervical deformities, and so may not be applicable to VAD patients with bony malformations. The last one is that we cannot avoid potential measurement bias.

## Conclusion

This is the first study to report the morphological characteristics and correlations of the pedicles and surrounding structures from C3 to C6 in patients with VAD. Smaller cervical pedicles were found from C3–C6 on the side with the dominant VA. POW < 4 mm and POW + DPVA < 5 mm were found most frequently at C3 and C4 in VAD patients, and CPS insertion is not recommended at pedicles with these characteristics. Dominant VA may affect indirectly POW, and TF may be a key determinant of DPVA and POW.

## Data Availability

The datasets in the current study are available from the corresponding author on reasonable request.

## Abbreviations

AVA: Area of vertebral artery
ATF: Area of transverse foramen
CPS: Cervical pedicle screw
CT: Computed tomography
CTA: Computed tomographic arteriography
DPVA: Distance from the lateral pedicle border to the closest part of vertebral artery
DVA: Diameter of vertebral artery
ICC: Intraclass correlation coefficient
IQR: Interquartile range
ORTF: Occupation ratio of transverse foramen
POW: Distance between the lateral cortical border and medial border at the most stenotic area of the pedicle
SD: Standard deviation
TF: Transverse foramen
VA: Vertebral artery
VAI: Vertebral artery injury
VAD: Vertebral artery dominance
